# Micro- and nano-fibers for organ-on-a-chip: Construction, applications, and prospects

**DOI:** 10.1016/j.mtbio.2024.101322

**Published:** 2024-10-31

**Authors:** Xiaoling Yang, Jingyan Shi, Bori Shi, Jianing Li, Chang Xue, Jingyun Ma, Xinghua Gao

**Affiliations:** a Materials Genome Institute, Shanghai University, Shanghai, 200444, China; b Ningbo Institute of Innovation for Combined Medicine and Engineering, The Affiliated Lihuili Hospital of Ningbo University, Ningbo, Zhejiang, 315040, China

**Keywords:** Organ-on-a-chip, Nanofiber membrane, Microfiber, Microfluidic spinning, Three-dimensional cell culture

## Abstract

Organ-on-a-chip, an in vitro biomimetic microsystem that enables precise regulation and real-time observation of the cell microenvironment, has the potential to become a powerful platform for recapitulating the real microenvironment of organs in vitro. Microenvironmental factors, such as living cells, three-dimensional (3D) culture, tissue–tissue interfaces, and biomechanical factors, are important cues in the construction of biomimetic microsystems. It is important to provide an appropriate 3D culture environment for living cells to grow. Fibers, particularly microfibers and nanofibers, can provide a suitable 3D culture environment for living cells via surface adhesion or internal loading. In addition, fibers can further expand their applications in tissue engineering and biomedical research by being assembled at a higher level in various ways to create functional 3D tissues or organs with more complex structures. The use of fiber to construct an organ-on-a-chip, whether as a 3D scaffold for cell culture or to more closely mimic real tissues/organs, will introduce new ideas and strategies for developing novel organ-on-a-chip systems. Based on this context, this review summarizes the research progress in the construction and applications of micro/nanofibers for organ-on-a-chip systems. It outlines the preparation methods and material selections for micro/nanofibers and provides a detailed overview of their respective strategies for cell 3D culture and organ-on-a-chip construction. This review also highlights the main research findings and applications of micro/nanofiber in this field, which have significant implications for future practice, and finally concludes by examining potential directions for future development.

## Graphical abstract

**Figure:**
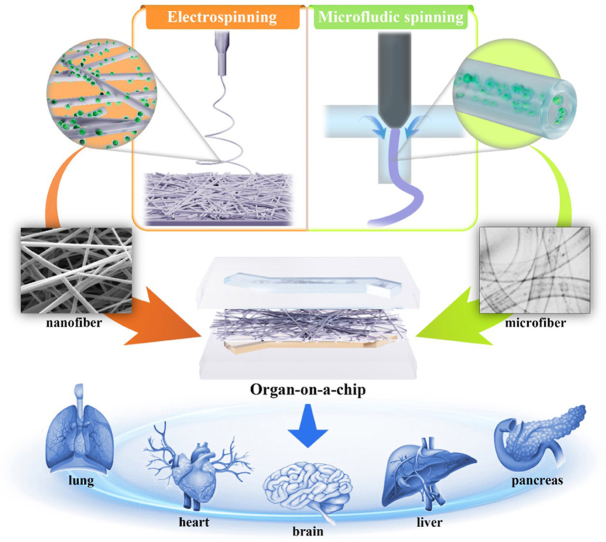

## 1 Introduction

Organ-on-a-chip is an in vitro biomimetic microsystem that has significant advantages in drug toxicity and efficacy evaluation compared to traditional models [1]. This microsystem integrates microchambers for cell culture and microchannels for fluid control, and can be used to simulate cell microenvironments and blood circulation systems (or other vessel systems) [2,3]. The organ-on-a-chip not only has the characteristics of a microfluidic chip that can be flexibly controlled and functionally integrated but also realizes precise regulation and real-time observation of the microenvironment, including biochemical factors (such as concentration gradients and cell–cell interactions) and biophysical factors (such as fluid shear and tension). Therefore, as a novel and integrated microsystem that can simulate the function of tissues and organs in vitro, organ-on-a-chip has the potential to become a powerful platform for remodelling the physiological structure and microenvironment of some tissues or organs in vitro [4,5]. With the advancements in biomaterials, tissue engineering, microfabrication, and microelectromechanical systems (MEMS), the development of organ-on-a-chip technology has gained much traction. Over the past two decades, the technological complexity and functionality of organ-on-a-chip have gradually increased, from simple chip design at the beginning of the use of three-dimensional (3D) printing for chip production and simple 3D cell culture to the simulation of human physiology and disease. Organ-on-a-chip is expected to be used in preclinical research and therapeutic testing [6]. Currently, many researchers have been exploring the construction of various organ-on-a-chip, mainly lung-on-a-chip [7], kidney-on-a-chip [8], gut-on-a-chip [9], and brain-on-a-chip [10]. Further, they have also obtained results in simulating pathological and physiological cell microenvironments, including COVID-19, renal fibrosis, intestinal microflora simulation, and blood–brain barrier construction [4].

Typically, in vitro biomimetic microsystems must abstract a biological model from the tissue/organ structure of the organism, analyze its numerous factors to establish a mathematical model, and then construct a technical model or device [11]. In this process, it is necessary to define parameters, optimize mathematical models, and then guide the construction of technical models to match the technical models with biological models, and finally match the structural characteristics of tissues/organs. It is generally accepted that the physiological function of a tissue or organ is only possible if it possesses the structural characteristics of the tissue or organ. Microenvironmental factors such as living cells, 3D culture, tissue interfaces, and biomechanical factors are key to constructing biomimetic microsystems. Providing a suitable 3D culture environment for living cells is essential. Currently, 3D cell scaffolds are widely used in cell 3D culture; they can fully preserve the specific phenotype and function of cells when cultured in suitable scaffolds with a spatial structure [12]. Furthermore, fibers can be assembled in high order by stacking, braiding, and 3D printing to obtain functional 3D structures with more complex results, such as microvascular networks [13], bio-printed muscles [14], cartilage tissue [15], and skin [16], and to further expand their applications in tissue engineering and biomedical fields. Based on the discussion above, the use of 3D scaffolds in cell culture not only provides a structural framework for cellular growth but also leverages their unique properties to enhance the alignment of 3D cell cultures with the structural characteristics of real tissues and organs. This approach facilitates the physiological recapitulation of tissues and organs in organ-on-a-chip models, offering new insights and strategies for the development of novel organ-on-a-chip systems.

Currently, the main preparation methods for fibers used as biological scaffolds include electrospinning and wet spinning [17]. Electrospinning is mainly used to prepare fibers with nanometer diameters. Because the cell size is usually on the micron scale, nanofibers are generally used in the form of nanofiber membranes for cell adhesion culture. Microfluidic spinning, which has been developed in recent years, is mainly used to prepare micron-sized fibers that can achieve more diverse cell culture models. These two types of fiber materials have been used to develop novel organ-on-a-chip systems.

**Fig. 1:**
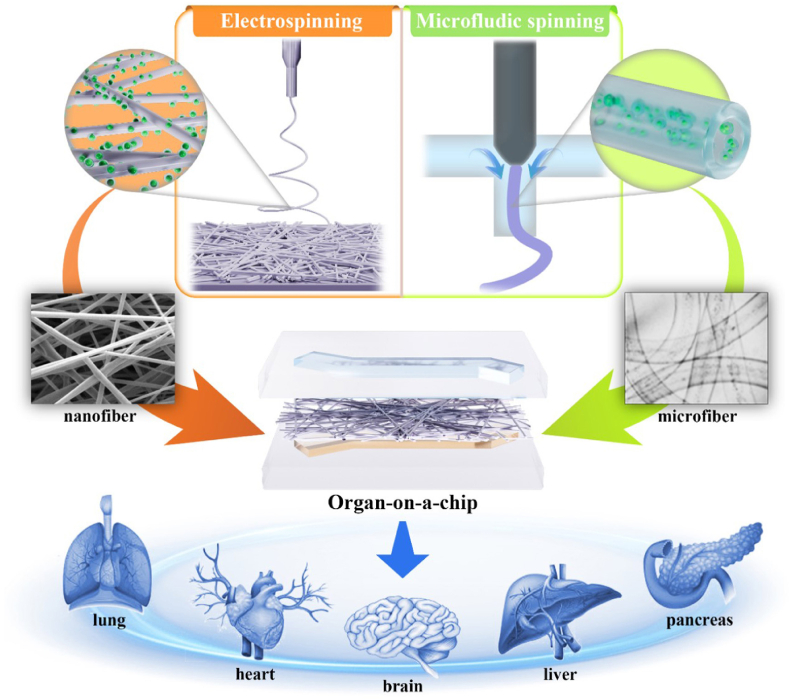
Schematic of micro/nanofibers prepared using electrospinning and microfluidic spinning for application in organ-on-a-chip.

This review describes the preparation methods and material selection of micro/nanofibers, and introduces their respective use strategies for cell 3D culture and organ-on-a-chip construction. Further, this review summarizes the application of micro/nanofibers in organ-on-a-chip, such as the lung, liver, heart, pancreatic islets, and multiple organs (Fig. 1).

## 2 Nanofiber membranes for organ-on-a-chip

### 2.1 Preparation and material selection of nanofibers

**Fig. 2:**
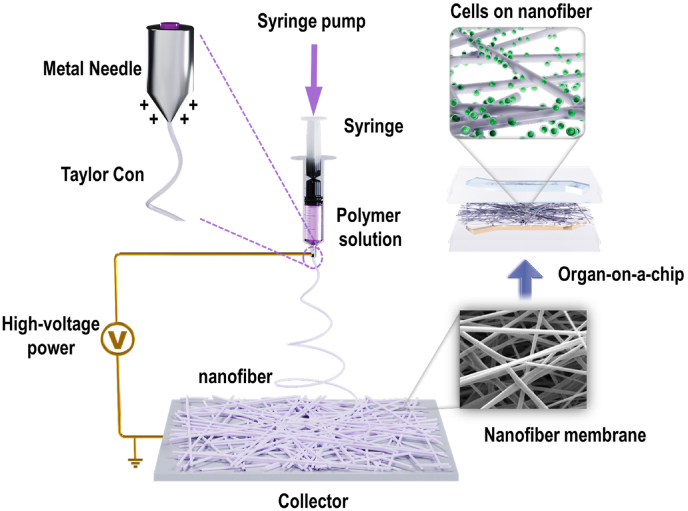
Schematic showing nanofiber membrane preparation via electrospinning and its usage strategies in organ-on-a-chip.

Owing to the diversity of nanofiber materials, their preparation methods are extensive. Electrospinning is one of the most widely used methods for preparing nanofibers. Electrospinning is a synthetic technology that produces nanofiber by jet-spinning a polymer solution under a high-voltage electrostatic field. The process involves a polymer solution being subjected to a strong electric field as it passes through a needle, causing its shape to change from spherical to conical, a form known as the “Taylor cone.” A jet stream is generated from the tip of this cone, which undergoes extension, deformation, solvent volatilization, and ultimately curing to form fine fibers. Electrospinning devices generally consist of four parts (Fig. 2): a syringe pump, syringe, high-voltage power supply, and collector [18,19]. The syringe needle is connected to the positive electrode of the high-voltage power supply, and the collector is connected to the negative electrode. The fiber collector can be a static plane, disk, or high-speed drum rotation. According to related research, the setting of spinning parameters, such as the spinning fiber materials and solvents, voltage, flow rate, receiving distance, environmental conditions, temperature, and humidity, are key factors during electrospinning [20].

The material selection for nanofibers depends on the requirements of a specific application. In the construction of organ-on-a-chip, nanofibers are primarily used as supporting materials for cell culture. Natural and synthetic polymers are the main materials used in electrospinning and have different characteristics. Natural polymers, including gelatin, collagen, hyaluronic acid, and chitosan are characterized by good biocompatibility and cell affinity, which contribute to cell attachment and growth. However, most natural polymers do not perform well in terms of their mechanical properties; it is difficult to construct organ-on-a-chip directly from natural fibers, and because of limited resources, they cannot be produced on a large scale. The main synthetic polymers used are polycaprolactone (PCL), poly (lactic-co-glycolic acid) (PLGA), poly L-lactic acid (PLLA), and polyurethane (PU). These synthetic polymers are generally biocompatible, biodegradable, and environmentally friendly and have a wide range of synthetic methods and sources for mass production. Researchers have also used mixtures of natural and synthetic polymers. For example, Bekezhankyzy et al. [21] used a mixture of PCL with a molecular weight of 45 kDa and collagen with a molecular weight of 300 kDa as raw materials to prepare nanofiber membranes. Compared with the pure PCL nanofiber membrane, the fibers formed in the PCL/collagen composite membrane were uniform in size, non-toxic, and had enhanced hydrophilicity and biocompatibility while retaining their elasticity appropriately, making them suitable for application in organ-on-a-chip.

### 2.2 Usage strategies

Owing to their unique structure and properties, nanofibers can accurately simulate the microenvironment of an organism on a nanometer scale and provide more suitable growth conditions for cell culture in vitro. Furthermore, nanofibers can be easily prepared to membranes with large specific surface area and porous structure, which enable cells to be seeded and grown on the surface of nanofibers. This type of membrane provides a nearly 3D culture environment for cells, which means that it is good for the exchange of nutrients and wastes and promotes cell adherence, proliferation, and differentiation [22]. Studies have shown that nanofiber membranes can be combined with microfluidic chips to study 3D cell cultures and differentiation. For example, Kim et al. [23] used electrospinning to prepare PCL nanofibers, which were placed at the bottom of the chamber of a polydimethylsiloxane (PDMS) chip. They seeded human hepatoma cells HepG2 onto the nanofibers and continuously perfused the culture medium to monitor the secretion of albumin and alpha-fetoprotein in real time. The results showed that HepG2 cells exhibited good activity and high proliferative ability after 14 days of culture, which could be used for drug evaluation. Jiang et al. [24] fabricated a 3D PLGA nanofiber membrane for culturing bone marrow mesenchymal stem cells (MSCs). Compared with the traditional two-dimensional culture cells, the MSCs on the nanofibers exhibited a 3D growth morphology, and the differentiation of MSCs on the nanofiber array was obvious in the osteogenically conditioned medium, indicating that the nanofiber could be used to simulate the bone microenvironment. Hesari et al. [25] also used PLGA nanofibers as substrates for microfluidic chips to culture human-induced pluripotent stem cells (hiPSCs). The results showed that hiPSCs adhered well to the PLGA nanofibers, and the expression of neuron-specific genes in the cells was significantly higher than that in the control group, indicating the successful differentiation of hiPSCs into neurons. The combination of nanofibers and 3D printing technology can improve the shape fidelity and cell compatibility of 3D printed cell scaffolds. For example, Zou et al. [26] combined 4 % RGD-containing *Antheraea pernyi* silk fibroin nanofibers (ASFNFS) (with partial natural silk structure) with 16 % pure gelatin as a new type of biological ink and 3D printed ASFNFS scaffolds. Compared with pure gelatin, the degradation and swelling rates of the scaffold were higher. Further, Schwann cells cultured on the scaffold also showed stronger vitality and proliferation. This study provided a reference for regulating other biological inks. Based on the above analysis, the combination of nanofiber membranes and microfluidic chips can provide a better microenvironment for cells, realize 3D cell culture in vitro, and promote cell–cell and cell–matrix interactions to construct a model of drug evaluation and the pathological/physiological microenvironment in vitro. By contrast, the environment of cells, tissues, and organs in the human body has a 3D structure. Organ-on-a-chip is a powerful platform to simulate the physiological structure and microenvironment of some tissues or organs in vitro, which means that, compared with the traditional 2D cell culture, it is also necessary to construct a 3D cell culture environment in vitro to simulate the tissue-to-tissue interface and use it to construct various types of in vitro models. This opens up new avenues for the application of nanofiber membranes.

### 2.3 Nanofiber membranes in lung-on-a-chip

Lung-on-a-chip is a typical organ-on-a-chip that mimics the interface between tissues. Various researchers have recapitulated the alveolar–capillary interface by culturing alveolar epithelial and endothelial cells on both sides of the PDMS microporous membrane. Furthermore, the cyclic tensile deformation of the PDMS membrane was controlled by air pressure to simulate a breathing lung and realize biomimetic construction of the lung in vitro. Nanofiber membranes have a high surface area and porosity, which make them more similar to the structure of the natural basement membrane in vivo [27]. Therefore, most studies on the combination of nanofibers and organ-on-a-chip have focused on the lungs (Table 1).

**Table 1:** Application of nanofiber membrane in organ-on-a-chip construction [21,23,[28], [29], [30], [31], [32], [33], [34], [35]].

Type of chip	Materials	Nanofiber/membrane dimensions	Cell Type	Point	Ref
Lung-on-a-chip	PCL; PCL/collagen	/	A549	PCL/collagen nanofiber is better than PCL nanofiber suitable for application in lung-on-a-chip.	[21]
	PLGA	Thickness: ∼ 3 μm.	A549 HFL1 HUVEC	As substrate and 3D scaffolds for co-culture; The evaluation of gefitinib and the mechanism of drug resistance.	[28]
	PLGA/PDMS	Thickness: PLGA ∼ 3 μm; PDMS ∼70 μm; micropores diameter: 200 μm	NCI-H1650 NCI-H460	Realize the deformation of the membrane; study the sensitivity of gefitinib.	[29]
	Gelatin	Porosity: >50 %; Size: 1∼10 μm; Thickness: 100∼500 μm.	A549	Simulate the periodic movement of lung respiration in vivo.	[30]
	PCL/gelatin	Thickness: ∼20 μm.	A549 HUVEC HSAEC	Study how the extracellular matrix structure affects epithelial cell injury during airway reopening.	[31]
	PU	Average diameter: 420 ± 99.43 nm; Average 2D pore size: 1.35 μm ± 436 nm.	HAECs HMVEC-Ls	After applying several different types of mechanical forces, the degree of cell damage was observed and the recovery of cells was monitored.	[32]
Heart-on-a-chip	PLLA; PU	Average diameter: PLLA 186 ± 65 nm; PU 318 ± 102 nm.	MSC HCM H9C2	Study the effect of MSCs on mitochondrial dysfunction in HCM and H9C2 cells.	[33]
Liver-on-a-chip	PCL	Diameter: 600–1000 nm; Membrane porosity: 76 %; Pore size: 3 μm; Thickness: 113.7 ± 2.7 μm.	HepG2	As a scaffold for cell culture; monitor the secretion of albumin and alpha-fetoprotein in real time.	[23]
Nerve-on-a-chip)	PLLA	Fiber densities:11 %, 46 %, 71 %	DRG	Study the impacts of nanofibers with different densities on the vertical velocity of axonal growth and the recovery of the axotomized areas.	[34]
multi-organ-on-a-chip	PLLA/collagen I	Average diameter: 325 ± 50 nm; Average pore size: <5 μm.	HFF LO2 PC-9 HUVEC	Simulate the tissue-tissue interface; establish the model of jaundice in vitro.	[35]

**Fig. 3:**
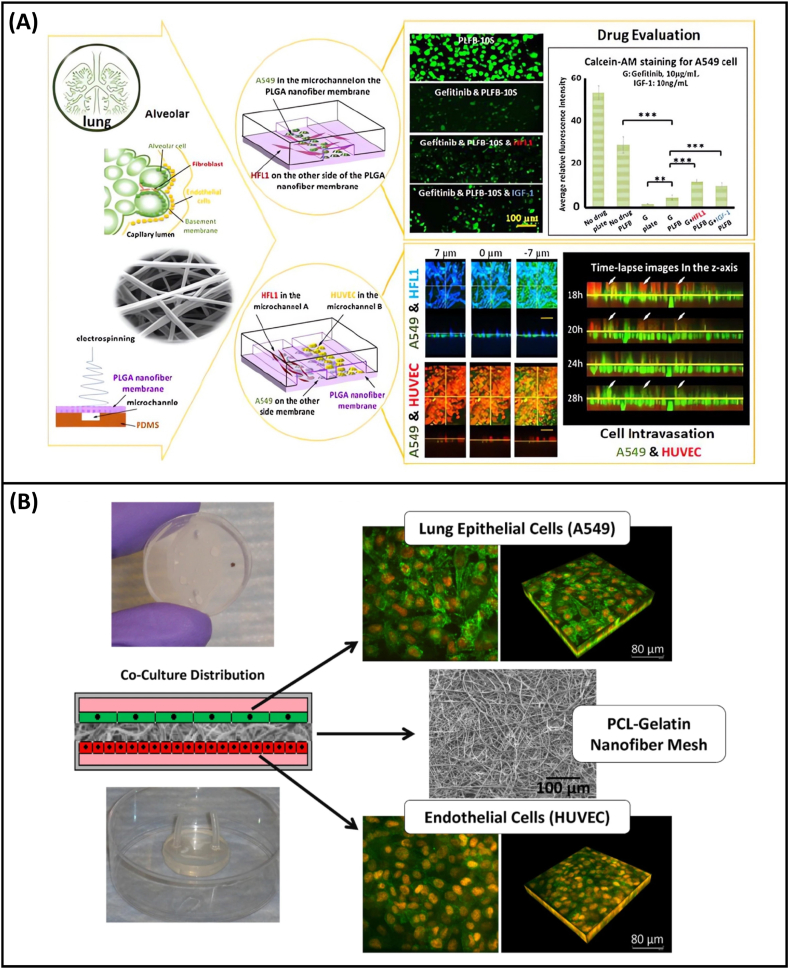
**Application of nanofiber membrane in organ-on-a-chip construction.** (A) Schematic of lung-on-a-chip design and development based on PLGA nanofiber membrane, and research on anti-cancer drug testing in a simulated alveolar microenvironment [28]; (B)Co-culture device and images of cells on PCL fiber network [31].

In 2018, Yang et al. [28] were the first to use PLGA to prepare a lung-on-a-chip based on nanofiber membranes. In this study, PLGA nanofiber membranes were prepared as substrates for a microfluidic chip and as 3D scaffolds for cell culture. Human non-small cell lung cancer cells A549, human fetal lung fibroblasts HFL1, and human umbilical vein endothelial cells (HUVECs) were co-cultured on the chip to evaluate the efficacy of gefitinib, an epidermal growth factor receptor (EGFR)-targeted drug, and to investigate the mechanisms underlying drug resistance in A549 cells (Fig. 3A). Because of its unique porous structure and molecular permeability, the PLGA nanofiber membrane can accurately simulate the alveolar respiratory membrane. However, because the PLGA nanofiber membrane was sealed with multiple layers of PDMS chips, this study failed to achieve elastic changes in the PLGA nanofiber membrane and could not simulate the respiratory effects of the lung. In a subsequent study, Li et al. [29] prepared a PLGA nanofiber and PDMS microporous composite membrane, succeeded in deforming the membrane, and studied the sensitivity of the lung adenocarcinoma cell line NCI-H1650 and the large cell lung cancer cell line NCI-H460 to gefitinib. In addition to the above studies on synthetic polymers, which used only PLGA to prepare nanofiber membranes for lung-on-a-chip, Radiom et al. [30] used the natural macromolecule gelatin as a raw material to prepare nanofibers, and a microchip simulating the alveolar air–tissue interface was developed. The single-layer gelatin nanofiber membrane was designed in a hexagonal shape and placed at the air–liquid interface to simulate the actual shape of alveolar air sacs. Then, by applying pressure, the stretching of the monolayer nanofiber membrane was realized, thus successfully simulating the periodic movement of lung respiration in a real human lung.

To further improve the mechanical strength and biocompatibility of nanofiber membranes, composite nanofiber membranes made of natural and synthetic polymers have also been used in lung-on-a-chip, PCL/collagen composite nanofiber membranes as mentioned above. Higuita-Castro et al. [31] used a composite of PCL/gelatin to prepare nanofiber membranes and co-cultured HUVECs with A549 cells or primary human small airway epithelial cells (HSAECs) to integrate a lung-on-a-chip (Fig. 3B). They investigated how the structural properties of the extracellular matrix affect epithelial cell injury and alveolar–capillary barrier properties when the airway is reopened. They found that the cells on the dense and hard PCL fiber membrane were more active, whereas the cells on the PCL/gelatin nanofiber membrane with the ratio of 1:1 were less damaged in the simulated airway reopening experiment. The cause of cell damage was found to be the surface tension that occurs when the alveolar reopens. This study provides a basis for the construction of a structure that is more closely related to the respiratory membrane of the lungs in vivo. This system can also be used to analyze the mechanisms of respiratory lung injury and other lung lesions. Gabela-Zuniga et al. [32] recently developed a new ventilator-on-a-chip (VOC) model based on the lung microenvironment to study the complex mechanisms of mechanical ventilation-induced lung injury and its subsequent recovery. In this study, human alveolar epithelial cells (HAECs) and human lung microvascular endothelial cells (HMVEC-Ls) were cultured and cocultured on PU nanofiber membranes. The cells can form vascular barriers and tight junctions on the PU nanofiber membranes. The degree of cell damage and recovery was observed by applying several different types of mechanical forces. This study confirmed that the VOC model can be used as a powerful platform for studying the mechanisms of respiratory lung injury.

### 2.4 Nanofiber membranes in other organ-on-a-chip

In addition to lung-on-a-chip, nanofiber membranes have been used in other organ-on-a-chip systems, including heart-on-a-chip, nerve-on-a-chip, liver-on-a-chip, and combined multi-organ-on-a-chip systems. For example, Kobuszewska et al. [33] selected PLLA and PU materials to prepare nanofibers and cultured cardiac cells on nanofiber membranes, which were then integrated into microfluidic systems to create a heart-on-a-chip. Researchers have used this chip to study the effects of MSCs on mitochondrial dysfunction in human cardiomyocytes (HCM) and rat cardiomyoblasts H9C2 metabolism. The nanofibers in the heart-on-a-chip promoted parallel arrangement of cardiac cells and MSCs. MSCs enhanced the metabolic activity of HCM and H9C2 cells, and the enhancement effect on HCM cells was greater. This heart-on-a-chip can be used to evaluate the effect of stem cells on pathological cardiac cells in vitro, providing a new approach for the treatment of heart transplantation and making it possible to simulate heart problems, such as heart failure, in vitro. Lee et al. [34] cultivated dorsal root ganglia (DRGs) on a peripheral nerve injury-on-a-chip (PNI chip) and assembled aligned PLLA nanofibers. The effects of nanofibers with different densities on the vertical velocity of axonal growth and recovery of axotomized areas were studied. The results revealed that, compared to the condition without nanofibers, the vertical velocity of axonal growth and the recovery area of the axotomized areas increased significantly when the nanofibers were arranged. The PNI chip is expected to serve as a powerful novel biomaterial evaluation and drug testing platform for the further optimization of PNI treatment. Lei et al. [35] proposed a multi-organ-on-a-chip based on PLLA/collagen I composite nanofibers for simulating tissue–tissue interfaces. The chip can be extended to integrate blood vessels, skin, liver, lungs, and other organs to establish in vitro jaundice models. They cultured human foreskin fibroblasts (HFF), liver cells LO2, and lung cells PC-9 in a chamber at the bottom of the chip, and HUVECs in the top chamber, thereby successfully constructing multi-organ-on-a-chip of blood vessel, skin, liver, and lung. The results showed that the cells had normal proliferative activity and responded to changes in bilirubin concentration. This study not only validated the feasibility of multi-organ-on-a-chip, but also demonstrated the potential of PLLA/collagen I-based nanofiber membranes for the interface simulation of various tissues and organ-on-a-chip. This type of nanofiber membrane is suitable for constructing many types of in vitro growth/pathology models, basic research, and drug development. This expands the applications of nanofiber membranes in the organ-on-a-chip field.

## 3 Functionalized microfiber for organ-on-a-chip

### 3.1 Microfluidic spinning and fiber material selection

**Fig. 4:**
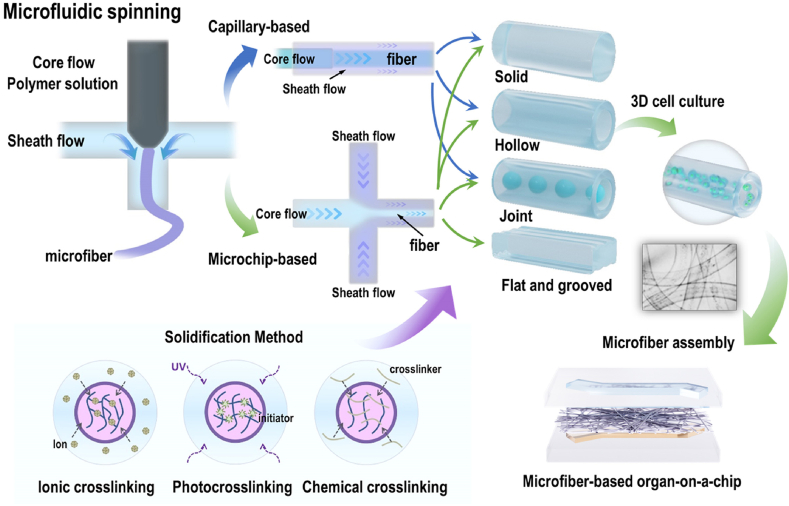
Schematic showing microfiber preparation via microfluidic spinning and its usage strategies in organ-on-a-chip.

Microfluidic spinning is a common method of preparing microfibers. As a novel fiber manufacturing method combining wet spinning and microfluidic technology, microfluidic spinning technology is suitable for preparing cell-loaded microfibers owing to its mild and rapid reaction conditions. Microfibers with controllable sizes, morphologies, structures, and compositions can be obtained by adjusting the fluid viscosity, flow rate, and microchannel design [36,37]. The preparation of microfibers by microfluidic spinning technology mainly uses a sample flow solution and a sheath flow solution with a certain viscosity to form a coaxial laminar flow in the microfluidic channel, which is then chemically or physically cross-linked and cured to obtain microfibers. In this process, the design of the microfluidic channel in the spinning system and the fluid perfusion parameters determine the shape and structure of the fiber, and the materials of the fiber determine the curing method and performance of the fiber, especially its biocompatibility. Therefore, in the microfluidic spinning process, the key elements of microfluidic channel design, fluid perfusion parameters, materials, and corresponding curing methods must be comprehensively considered. The spinning method is related to the design of microfluidic channels and the parameters of fluid perfusion. According to the different methods for constructing microfluidic channels, microfluidic spinning can be divided into the capillary nesting method and microfluidic chip method (Fig. 4).

Capillary nesting can be used to construct microfluidic channels by increasing the number of nested capillaries and changing their arrangement. For example, Yu et al. [38] prepared solid, core-shell, or spindle-knotted multi-structure and multi-component microfibers based on single-, double-, and multi-tube capillary nesting methods using alginate and GelMA materials, respectively. The advantages of this method are the ease of preparation of the glass capillary, its low cost, controllable size, and good hydrophilicity. These advantages facilitate the formation of a stable coaxial laminar flow. In addition, glass is resistant to acidic and organic solvents, making it compatible with a variety of materials for preparing fibers, further expanding its application scope. However, this method has some limitations. First, the glass capillary is fragile, and the nesting process requires highly skilled operation and precise equipment alignment. Second, the resulting fiber structure is usually relatively simple; if more complex microfibers are required, higher precision in capillary nesting and improved performance of the perfusion connection equipment are necessary. This increases the operational complexity, extends the time required, and reduces the overall efficiency. In contrast, the microfluidic chip method can accurately prepare functionalized microfibers with various morphologies and doping by simply adjusting the number and size of microchannels and combining advanced methods such as microvalve and microdroplet technology. In particular, the application of 3D printing technology to prepare microfluidic chip templates has further reduced the difficulty of microfluidic chip preparation. The introduction of this technology not only simplifies the preparation process but also provides a unique opportunity to synthesize fibers with customizable geometric and chemical complexities. Yu et al. [39] combined the microfluidic droplet technology with the spinning process to prepare hybrid bamboo-like calcium alginate microfibers. Furthermore, hollow microfibers with multi-chamber and multi-component structures were prepared by varying the number of chip layers and the microchannel structure [40]. Yao et al. [41] successfully prepared four-component heterogeneous calcium alginate microfibers with different arrangements using a three-layer PDMS sandwich microchip. Li et al. [42] prepared microchip templates with different heights using a one-step 3D printing technology. Based on these templates, they fabricated microchips that enabled the preparation of single-component/single-hole, two-component/two-hole, and two-component/three-hole calcium alginate microfibers.

In addition to the aforementioned spinning methods, the selection of fiber materials and corresponding curing methods should be carefully considered when functionalized microfibers are used as biological scaffolds. The materials used in microfluidic spinning can be divided into synthetic and natural polymer materials. Synthetic polymer materials include 4-hydroxybutyl acrylate (4-HBA) [43], polyethylene glycol diacrylate (PEGDA) [44], and PCL [45]. Most of these materials exhibit good biocompatibility, degradability, and mechanical properties. These are biopolymer materials commonly used in tissue engineering; however, their hydrophilicity is limited. Natural polymers include alginate, hyaluronic acid, chitosan, and collagen. Natural polymers are various natural extracts with excellent biocompatibility and degradability, and are widely used to simulate extracellular matrix components. However, compared to synthetic polymer materials, the mechanical properties and stability of natural polymer materials are poor, which limits their application as tissue interfaces and cell culture scaffolds [46,47]. Among these, alginate is the most commonly used material. As a natural polysaccharide extracted from algae (such as brown algae), alginate can be dissolved in water to form a viscous liquid and undergo a rapid cross-linking reaction, which is suitable for microfluidic spinning systems in aqueous solution environments [48]. In addition, to better meet the needs of biomedical applications, researchers typically further compound or modify the materials. For example, preparing microfibers using a double-cross-linked composite material composed of GelMA and alginate can enhance the activity of adherent cells [49,50]. Simple chemical cross-linking modifications of hyaluronic acid can improve the hydrophilicity and mechanical properties of the fiber materials [51]. Additionally, modifying GelMA or alginate microfibers by covalently linking various peptide sequences (such as RGD) can promote cell adhesion to the fibers [52,53].

Microfluidic spinning requires different cross-linking and curing methods for different materials, e.g., ion cross-linking, photo cross-linking, and chemical cross-linking (Fig. 4). Ion cross-linking is the most common type of cross-linking reaction in microfluidic spinning. The basic principle involves utilizing the rapid cross-linking reaction between the sample flow and sheath flow to achieve fiber curing. For example, an alginate solution is typically used as a sample flow, and a divalent cation (such as Ca^2+^, Ba^2+^, Sr^2+^, Cd^2+^, and Zn^2+^) solution is used as a sheath flow, which can coordinate with these divalent cations to form microfibers rapidly. Among these cations, Ca^2+^ is widely used because of its non-toxicity and moderate affinity for alginates. However, ion cross-linking between alginates and Ca^2+^ is relatively weak, and other ions in the physiological environment can exchange with Ca^2+^ in the calcium-alginate hydrogel, resulting in the decomposition of calcium alginate in the physiological environment owing to the loss of Ca^2+^. Photo cross-linking is another method for preparing microfibers, which mainly involves a polymer monomer and photo initiator. In this method, a sample flow from the outlet to stabilize the flow, followed by UV exposure, resulting in polymerization and the formation of microfibers. This method is particularly suitable for polymer materials that require photo cross-linking curing, such as GelMA and PEGDA. Chemical cross-linking involves the addition of a cross-linking agent to the sheath flow solution for diffusion into the sample flow solution, which occurs when the two are in contact with each other, thereby achieving the formation of microfibers. In general, ion cross-linking and photo cross-linking are simple and efficient curing methods that are commonly used in microfluidic spinning.

### 3.2 Usage strategies

Microfluidic spinning provides mild conditions for preparing microfibers, and the materials used have good biocompatibility. Owing to their high porosity, controllable structural direction, large specific surface area, and diverse mechanical properties, the materials used for preparing microfibers are suitable for use as scaffold materials for cell culture. There are usually three cell culture methods: first, cells can be cultured on the surface of microfibers; second, cells can be encapsulated within microfibers; and third, when hollow microfibers are produced, cells can be cultured on the inner surface of the hollow lumen. It is relatively easy to culture cells on the surface of microfibers. Compared with nanofibers, microfibers have a larger surface, which is comparable to or slightly larger than the cell size, enabling cells to adhere to and grow on a single fiber. In addition, by changing the geometry of the cross-section of the fluid channel, microfibers with complex structures, such as grooves, pores, and flats, can be obtained, and cells can be cultured on them to control their arrangement and orientation. Zhao et al. [54] prepared GelMA/calcium alginate composite microfibers with grooved surfaces and cultured murine skeletal myoblast C2C12 cells on the microfibers. The experimental results showed that the cell activity was good and cells only grew in the groove part of the microfiber; the C2C12 cells were elongated along the groove, indicating that the grooved microfiber had good biocompatibility and cell orientation. Patel et al. [55] and Sharifi et al. [45] studied the proliferation and differentiation of adult hippocampal stem/progenitor cells (AHPCs) using PCL microfibers with different topological structures and sizes as scaffolds. AHPCs can preserve the multipotential differentiation ability of the PCL scaffolds. Following differentiation, oligodendrocytes and astrocytes align along the axial direction of microfibers, and the expression of early neuronal markers (β-tubulin) and neuronal dendritic markers (MAP2ab) increases significantly. Haynl et al. [56] prepared collagen microfibers and cultured neuronal NG108-15 cells on these microfibers. The cells aligned along the axial direction of the microfibers and exhibited axon growth, with axon lengths reaching up to 100 μm. These studies show that culturing cells on the surface of microfibers, especially those with special structural surfaces, can promote the orientation or functional expression of cells. This discovery has significant potential for the 3D culture of nerves, muscles, and other tissue cells in vitro, as well as for the development of organ-on-a-chip systems.

In addition to cell culture on the surface of microfibers, microfibers can also achieve cell encapsulation, i.e., the cells are mixed into the sample flow. After the cross-linking reaction, the microfiber serves as a scaffold material to provide a 3D culture environment for the cells, thereby maintaining the specific phenotype and function of the cells. To ensure that the activity of the cells is not affected during the microfluidic spinning process, an ion cross-linking system of alginate-calcium chloride or a photo cross-linking system of a water-soluble initiator is typically selected. For example, Onoe et al. [57] prepared hydrogel microfibers with calcium alginate as the shell and natural extracellular matrix proteins (such as fibrinogen and collagen), and cell mixtures as the fiber core. This approach realized the encapsulation of various cells, including endothelial cells, cardiomyocytes, and nerve cells. Additionally, these microfibers can be assembled into more complex structures through winding or weaving, thereby meeting the requirements of tissue engineering. Yamada et al. [58] used an alginate solution mixed with hepatocytes and the mouse embryonic fibroblast cell line Swiss 3T3 as a sample flow to prepare microfibers based on a multichannel PDMS microchip. This method allowed hepatocytes to be encapsulated by Swiss 3T3 cells in the middle of fibers, thereby mimicking the hepatic cord structure in the liver. The results showed that the cells retained high cell activity after being cultured for more than 30 days (approximately 80 % survival rate). Yao et al. [41] simulated the lung tumor microenvironment by a multi-day co-culture of HFL1, NCI-H1650, and HUVECs in calcium alginate microfibers and studied the intercellular interactions during the development of lung tumors. Huan et al. [59] prepared hydrogel microfibers with a double network structure loaded with islet α and β cells by using the microfluidic spinning technology. They used alginate and methacrylated hyaluronic acid (Alg-HAMA) as raw materials to prepare the microfibers. They encapsulated the islet α cell line (α-TC) and β cell line (MIN-6) within hydrogel microfibers. The survival and proliferation ability of these cells were found to be higher than those of the control group, and they were able to secrete insulin and glucagon normally. This indicates that the hydrogel microfibers prepared by microfluidic spinning have higher biocompatibility.

Hollow microfibers have a unique hollow structure, and a special hollow cavity can be obtained by improving the nozzle structure. The preparation of hollow microfibers occurs mainly through the introduction of an inert fluid as the core flow in microfluidic spinning to form a placeholder, thereby obtaining hollow microfibers. Hollow microfibers are structurally capable of mimicking lumen-like structures such as blood vessels, the trachea, cardiac fibers, and nerve bundles, which are of great significance for tissue engineering and biomedical research. The blood circulation system is closely related to all organs in the body and is responsible for transporting cells, nutrients, growth factors, and oxygen, as well as removing metabolic waste. Therefore, in vitro vascular simulations are crucial for the construction of bionic tissue and organ models. Lee et al. [60] prepared hollow calcium alginate microfibers loaded with human iliac vein endothelial cells (HIVE-78) using microfluidic spinning. The cell-loaded microfibers were embedded in an agar-gelatin-fibronectin hydrogel loaded with smooth muscle cells to simulate vascular tissue. The resulting hydrogel maintained its functional vascular structure for 7 days. Bosch-Rue et al. [61] prepared double-layered hollow microfibers. A mixture of alginate/collagen I from human aortic smooth muscle cells (HASMCs) formed the outer layer of the fiber, and collagen I mixed with HUVECs formed the inner layer of the fiber. After 20 days of culture, the cell survival rate was higher than 90 %, and the distribution of cells in each layer was similar to the physiological arrangement in vivo. Inspired by the different geometric shapes of blood vessels in vivo, Jia et al. [62] prepared cell-loaded calcium alginate/collagen composite hydrogel spiral hollow microfibers. The fibers showed good perfusion and simulated a spiral vascular structure and blood flow. Hydrogel microfibers can support the growth of HUVECs on the inner walls of hollow microfibers and promote their development into luminal structures.

Similarly, Guo et al. [63] used HUVECs suspension as the core flow to prepare hollow bulb-shaped microfibers containing endothelial cells using the microfluidic chip method. The structure was highly similar to that of irregular blood vessels (such as varicose veins), and an in vitro varicose vein disease model was established. In addition to the above-mentioned construction based on hollow microfiber blood vessels, the use of double- or multi-lumen hollow microfibers to construct blood vessels and tissue complexes can better simulate physiological and pathological microenvironments and construct a new in vitro model. For example, Yu et al. [40] prepared hollow microfibers with multiple compartments or components using microfluidic chip spinning. Human primary glioblastoma cell lines U87 and HUVECs were separately encapsulated and co-cultured without contact to explore the progression of glioma and tumor vascularization in the tumor microenvironment. Li et al. [42] encapsulated normal pulmonary bronchial epithelium cells 16HBE and HUVECs as two different components in two-component two-pore microfibers to simulate the bronchial lumen and blood vessels of the lung. Wei et al. [52] prepared double-layer hollow microfibers loaded with human osteoblast-like MG63 and HUVECs, which formed bionic bone-like structures. Microfibers that promote angiogenesis and osteogenic expression can easily be assembled into complex tissue structures.

These studies show that the preparation of hollow microfibers based on vascularization has potential applications for simulating complex tissues or organs. Currently, microfluidic channels are typically used to simulate blood vessels in an organ-on-a-chip; however, the interfaces of these microchannels are mostly rectangular, which makes it difficult to ensure that all channel walls are vascularized. To overcome this limitation, hollow microfibers can be used to construct a more realistic vascular network on organ-on-a-chip, thereby developing a new organ-on-a-chip model.

### 3.3 Application of microfibers in organ-on-a-chip construction

The vascular system is responsible for various physiological and pathological processes in all organs of the body, transporting cells, nutrients, growth factors, and oxygen and removing metabolic waste. Hollow microfibers exhibit excellent performance in simulating lumen-like tissues mainly because they are structurally similar to the lumen structure, can achieve fluid perfusion, and have a large specific surface area, which is more conducive to material exchange. Therefore, hollow microfibers can be used to simulate blood vessels in vitro or to construct other lumen-like tissues and study the effect of perfusion on cell behavior to construct a new organ-on-a-chip.

**Fig. 5:**
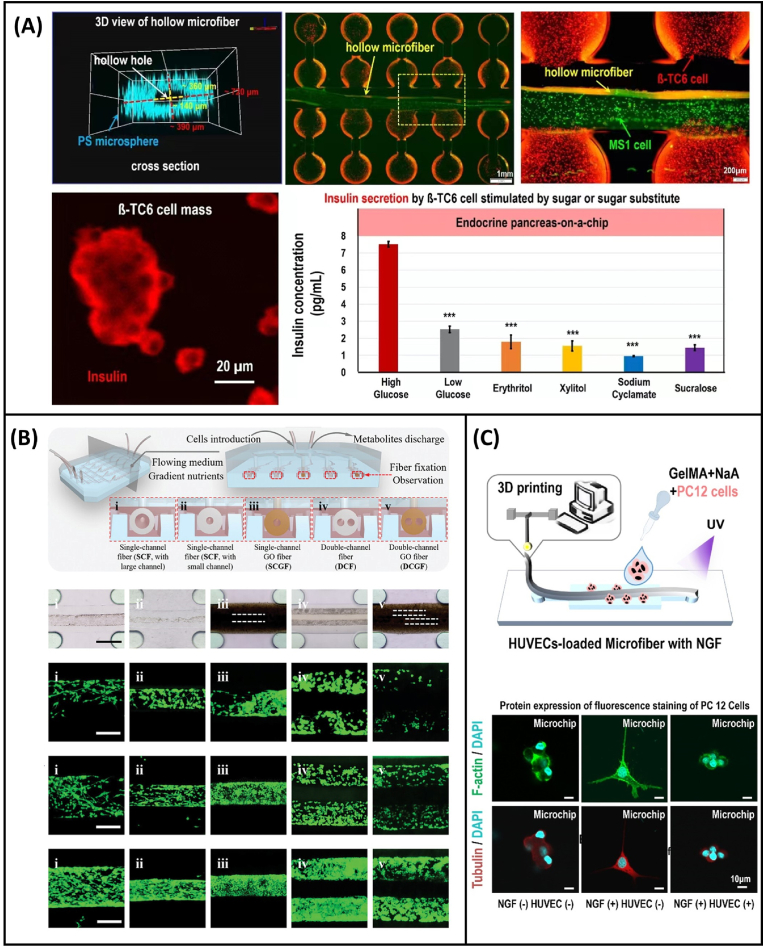
**Application of microfibers in organ-on-a-chip construction.** (A) Construction of pancreas-on-a-chip using a hollow microfiber assembly loaded with cells, and its application in evaluating sugar substitutes [65]; (B) schematic showing a nerve-on-a-chip, illustrating the proliferation of cells on microfibers with different structures [66]; (C) schematic of the neural model, depicting the expression of F-actin and tubulin in PC12 cells under different conditions [67].

Because microfibers can be used for cell culture in different ways as discussed above, different types of chips can also be developed by combining them with the organ-on-a-chip technology (Table 2). Lee et al. [64] prepared chitosan microfibers based on microfluidic spinning, successfully seeded HepG2 cells onto the surface of the microfibers to construct a liver organ-on-a-chip, and evaluated the effect of the microfibers on liver tissue formation. They analyzed the function of liver cells cultured on the surface of the chitosan microfibers by measuring urea synthesis and albumin secretion. The results showed that cell-loaded microfibers could be used as a scaffold for constructing liver tissue in vitro and for liver organ-on-a-chip in liver disease-related research. It can also be used in liver tissue engineering. Tian et al. [65] recently developed a novel islet endocrine organ-on-a-chip based on GelMA/calcium alginate composite hollow microfiber assembly. They first prepared mouse islet endothelial cell (MS1)-loaded GelMA/calcium alginate composite hollow microfibers using microfluidic spinning to simulate the vascular lumen structure and achieve vascularization. Subsequently, the cell-loaded microfibers were assembled with the islet 3D culture layer to construct a new islet organ-on-a-chip. After islet function reconstruction and analysis, the effects of glucose and sugar substitutes (erythritol, xylitol, sodium cyclamate, and sucralose) were evaluated in vitro (Fig. 5A). This islet endocrine organ-on-a-chip expands the application of organ-on-a-chip in the field of food safety and its application range. It can also be used to construct physiological models of islet diseases and for in vitro drug evaluation.

**Table 2:** Application of microfibers in organ-on-a-chip construction [[64], [65], [66], [67]].

Type of chip	Materials	Fiber manufacturing method and size	Cell type	Cell loading method	point	Ref
Liver-on-a-chip	Chitosan	Capillary-based; Diameter:70–150 μm.	HepG2	The surface of microfibers	As cell scaffolds to evaluate the effect of microfibers on liver tissue formation.	[64]
Nerve-on-a-chip	Alginate GelMA	Capillary-based; Diameter: 200/400 μm.	Schwann cell	The inner wall of hollow microfibers	Study the relationship between the proliferation rate of nerve cells in microfibers.	[66]
Islet-on-a-chip	Alginate GelMA	Microchip-based; Cross section: 390–720 μm Cavity: 140–360 μm	MS1 β-TC6	The inner wall of hollow microfibers	Achieve vascularized and evaluated sugar-substituted on insulin and glucagon secretion.	[65]
Nerve-on-a-chip	Alginate GelMA	Microchip-based;	HUVEC PC12	The inner wall of hollow microfibers	Construct a neural model based on the HUVEC-loaded hollow microfibers.	[67]

Yu et al. [66] proposed a new type of nerve-on-a-chip. They prepared GelMA/calcium alginate composite hollow microfibers by capillary nesting, and integrated the microfibers with Schwann cells and extracellular matrix into a chip with a multi-channel structure to form a nerve-on-a-chip. Studies have shown that the proliferation rate of cells in microfibers is correlated with the concentration of the extracellular matrix. In other words, the increase in extracellular matrix concentration promotes the proliferation of nerve cells and the formation of cell bundle arrangements in hollow microfibers (Fig. 5B). The microfibers in the chip were used in a rat sciatic nerve injury model to demonstrate that this nerve-on-a-chip can screen and evaluate microfibers that are effective for nerve fiber regeneration and functional recovery. This indicates that the organ-on-a-chip can open up a new evaluation approach for the application of biological scaffolds in tissue engineering in vivo. Similarly, Ma et al. [67] implemented different flow injection strategies on a chip and successfully obtained GelMA/calcium alginate composite microfibers with different numbers of hollow lumens via microfluidic spinning. They constructed a nerve-on-a-chip based on hollow HUVEC microfibers and rat adrenal medullary pheochromocytoma cells (PC12). They used the nerve growth factor to induce neural differentiation of PC12 cells. Axon length, tubulin, GAP-43, and TH-related gene expression in PC12 cells were detected (Fig. 5C). The construction of this in vitro neural model is expected to provide a new platform for studying nervous system diseases and evaluating new drugs and toxins. It has wide application prospects in the construction of organ-on-a-chip and blood–brain barrier simulations in vitro. In addition, to further expand the application of microfibers in the development of organ-on-a-chip systems, Park et al. [68] used the microfluidic technology to simultaneously spin multi-strands of “noodle-like” fibers with adjustable sub-micron thickness, creating noodle films. They conducted experiments to verify the ability of these fibers to act as independent porous films, which included diffusion tests and cell culture tests using the fibroblast cell line L929 and primary neural stem/progenitor cells (NSPCs). The results showed that the porous membrane had an obvious molecular diffusion ability, and the cells adhered well to the membrane, indicating that the noodle membrane could be used as a porous membrane. This membrane can potentially be used in the development of various types of organ-on-a-chip clips.

## 4 Challenges and prospects

Organ-on-a-chip research can be traced back to 2004. In 2011, the U.S. government launched a human organ-on-a-chip program. In 2015, Nature commented that the organ-on-a-chip is a revolutionary technology that could replace animal experiments in the future [69]. In 2016, the Davos World Economic Forum ranked it among the “top ten emerging technologies.” Twenty years ago, we witnessed the process of organ-on-a-chip development, from concept to emerging development. This emerging technology continues to intersect and integrate with materials science, chemistry, biomedicine, engineering, and other disciplines, fostering innovation and breakthroughs across various fields. As highlighted in the previous discussion, the application of fiber materials in the field of organ-on-a-chip is still in its early stages, and numerous challenges remain in various aspects. Moreover, it is essential to integrate this technology with other technologies to enhance its effectiveness and applicability.

(1) The selection of fiber materials indicates that the natural polymer or synthetic polymer materials used to prepare micro/nanofibers are still relatively limited. Owing to the complex microenvironments of different tissues/organs in the body, a single material cannot meet all the requirements of organ-on-a-chip. Since the introduction of the Material Genome Project was put forward, researchers have realized the crucial role of material innovation in technological development and industrial advancement. Consequently, the design and analysis of polymer materials have been progressing steadily. Currently, existing research only adopts the “trial method,” which involves a process of “trial” or “trial and error” for specific materials. Notably, in the context of microfluidic spinning, the alginate-calcium chloride system appears to be the most stable and repeatable, whereas other systems often represent modifications or improvements of this system. Recently, researchers have introduced nanomaterials or materials with nanostructures into fibers to form nanocomposite fibers, which are composite fibers with specific functions. Nanomaterials can endow fibers with corresponding functional properties such as conduction, drug loading, and storage. In the future, a combination of nanocomposite fibers and organ-on-a-chip in vitro could be realized, and a bionic system with certain functions could be constructed [70]. The potential breakthroughs in material design and computation in the field of biology are likely to remain a research hotspot at the intersection of materials science and organ-on-a-chip technology for the foreseeable future. Experimental testing or in-depth exploration through material design will offer limitless possibilities for identifying fiber materials that meet specific requirements. In addition, the integration of synthetic biology and materials science can significantly advance the development of materials science from two perspectives: material properties and material production methods, which provides new technologies for the development of new materials. Simultaneously, the study of biomaterials also presents a novel avenue to expand the field of synthetic biology.
(2) The application strategy of fiber materials in organ-on-a-chip systems involves using these materials primarily as membrane components to seal the chip, thereby creating interfaces between tissues. Additionally, fiber materials are utilized as scaffolds for 3D cell culture, filling the culture microchambers within the chip. The former presents new requirements for sealing technology and the mechanical properties of fiber membranes, whereas the latter does not fully exploit the advantages of the fiber structure and function. Recently, there has been an increasing research on the preparation of composite fiber membranes to enhance their mechanical properties, making them suitable for multi-type organ-on-a-chip, which may offer a viable solution. Further, some researchers suggest that the fiber material preparation technology based on microfluidic spinning can be reintegrated with the 3D bioprinting technology. The application of bio-3D printing technology in the fabrication of fibers and organ-on-a-chip systems may facilitate the development of novel organ-on-a-chip models and promote the construction of micro-physiological systems and organ remodelling in vitro. Electrospun fibers can be utilized in flexible bioelectronics to create flexible sensors that enable real-time monitoring and responses to biological signals. If electronic fibers can be successfully prepared and integrated into an organ-on-a-chip system, allowing for effective real-time feedback of biochemical signals through the sensor, it would represent a significant advancement in organ-on-a-chip technology. However, the biocompatibility and stability of the sensors and fibers remain critical factors that must be considered [71].
(3) The development of organ-on-a-chip also presents new challenges for the integration of fiber materials within chips. Currently, research in this field has begun to explore multi-organ joint chips and organoid chips [72]. In the study of fiber membranes for organ-on-a-chip systems, we have observed the potential applications of fiber materials in multi-organ joint chips. However, for organoid chips, existing fiber materials will introduce new requirements for biocompatibility and their adaptability to stem cells, particularly to induced pluripotent stem cells. A significant challenge will be determining whether the unique structure and function of fiber materials can effectively guide the proliferation and differentiation of stem cells in organ-on-a-chip system, promoting the development and maturation of organoids.

The application of micro/nanofibers in organ-on-a-chip technology is both a challenge and an opportunity, and many technical difficulties must be overcome. However, the addition of micro/nanofibers provides a new approach for constructing an organ-on-a-chip. With the continuous advancement of research technologies and the deepening of scientific research, it is believed that existing challenges will gradually be overcome. This progress will foster innovation and development in the field of organ-on-a-chip, propelling it to a new stage.

## CRediT authorship contribution statement

**Xiaoling Yang:** Writing – original draft, Investigation, Conceptualization. **Jingyan Shi:** Writing – original draft, Investigation. **Bori Shi:** Visualization, Investigation. **Jianing Li:** Validation, Investigation. **Chang Xue:** Validation, Investigation. **Jingyun Ma:** Writing – review & editing, Project administration, Funding acquisition. **Xinghua Gao:** Writing – review & editing, Visualization, Supervision, Project administration, Funding acquisition.

## Declaration of competing interest

The authors declare that they have no known competing financial interests or personal relationships that could have appeared to influence the work reported in this paper.

## Data Availability

No data was used for the research described in the article.
